# Reduction of tumor volume during radiotherapy in patients with small-cell lung cancer and its prognostic significance

**DOI:** 10.1007/s00066-023-02146-x

**Published:** 2023-09-21

**Authors:** Christian Kandler, Khaled Elsayad, Georg Evers, Jan Siats, Christopher Kittel, Sergiu Scobioala, Annalen Bleckmann, Hans Theodor Eich

**Affiliations:** 1 Department of Radiation Oncology, University Hospital Muenster, Albert-Schweitzer-Campus 1, Building A1, 48149 Muenster, Germany; 2 Department of Medicine A (Hematology, Oncology, Hemostaseology and Pulmonology), University Hospital Muenster, Muenster, Germany

**Keywords:** Small-cell lung cancer, Radiotherapy, GTV changes, Radiomic biomarker, Tumor volume

## Abstract

**Background:**

Several studies have reported the potential prognostic significance of tumor volume reduction ratio (VRR) induced by radiotherapy (RT) in patients with non-small-cell lung cancer. However, there are no data yet on the prognostic significance of volumetric shrinkage in patients with small-cell lung cancer (SCLC). This study aimed to demonstrate the correlation between tumor volume reduction ratio and treatment outcomes.

**Materials and methods:**

The study included 61 patients with SCLC treated with fractionated RT of the primary tumor at our institution between 2013 and 2020. The relationship between volumetric changes in gross tumor volume (GTV) during radiotherapy and outcomes were analyzed and reported.

**Results:**

The median radiation dose was 59.4 Gy (median fraction dose was 1.8 Gy). The median GTV before radiotherapy was 74 cm^3^, with a median GTV reduction of 48%. There was a higher VRR in patients receiving concurrent radiochemotherapy (*p* = 0.05). No volumetric parameters were identified as relevant predictors of outcome in the entire cohort. In multivariate analysis, only age had an impact on survival, while prophylactic whole-brain radiation influenced the progression-free survival significantly.

**Conclusion:**

Concurrent chemotherapy was associated with a higher VRR than sequential chemotherapy. No significant impact of VRR on patients’ outcome or survival was detected.

## Background

Small-cell lung cancer (SCLC) represents approximately 10–15% of all lung cancer types. The rapid doubling time, genomic instability, and increased vascularity lead to fast tumor growth with an early development of disseminated metastases, making SCLC the most aggressive type of lung cancer. Lung cancer development is the standard typically with cigarette consumption and low socioeconomic position regarding education, occupation, and income. Historically, SCLC has been classified according to the two-stage system established by the Veterans’ Administration Lung Study Group [1]. This classification focused primarily on the feasibility of radiotherapy (RT) for the primary tumor confined to one hemithorax and distinguishing between limited and extensive disease. Limited-stage patients are those whose disease is confined to one hemithorax and regional lymph nodes, with no disease outside the chest. Extensive-stage disease includes all other patients whose disease has spread beyond the limited-stage disease. The system has since been expanded to include an additional stage (very limited disease) incorporating the current TNM characteristics and is still used in most clinical trials today. At initial diagnosis, approximately 5% of patients have a very limited stage disease, 30% of patients have a limited-stage disease. The remaining patients are already in the extensive-stage disease. The median survival time reaches 20 months in patients with limited disease and appropriate treatment. On the other hand, the median survival time for an untreated disease is less than 3 months. With appropriate treatment strategies, the median survival time for patients with untreated distant metastases is reaching 12 months [2–7].

According to the current guidelines, concurrent platinum-based radiochemotherapy (RCT) with subsequent prophylactic whole-brain irradiation (WBI) is regarded as standard treatment for SCLC patients with limited disease [8, 9]. At this stage of disease, a recent study has shown that in patients treated with thoracic RT and WBI, intratherapeutic maximal serum lactate dehydrogenase levels are predictive of possible brain metastasis and survival [10].

Consolidation immunotherapy after RCT has not yet shown any benefit in progression-free survival (PFS) or overall survival (OS) for patients with limited-stage disease [11]. The addition of immunotherapy (anti-PDL‑1; atezolizumab or durvalumab) to chemotherapy (CTx; platinum-based) improves OS in the treatment of extensive-stage SCLC. Furthermore, for a minority of eligible patients with very limited disease stage, surgical resection followed by adjuvant treatment is a feasible treatment option, particularly in nodal-negative patients [12–16]. Recent studies have also investigated the safety and long-term control of stereotactic RT of primary lung cancer [17]. Concurrent RCT is the established standard of care for patients with limited-stage SCLC. For patients with extensive-stage disease, the standard of care includes medical therapy with CTx and immunotherapy. In cases where the primary tumor is amenable to radiation, RT is used as a consolidation therapy and for symptom relief as an individual decision. It is recommended that CTx be initiated promptly after diagnosis in all stages, using combination CTx. The optimal schedule and dose of RT in the management of SCLC remains a subject of ongoing debate. As a result, ongoing trials continue to compare hyperfractionated accelerated RT with conventional fractionated RT [18, 19].

Unfortunately, despite all therapeutic approaches, SCLC tends to recur within the radiation field and metastasize to distant sites. Modern radiation techniques, such as intensity-modulated radiotherapy (IMRT) and image-guided radiotherapy (IG-RT), are usually used to spare the heart and nearby normal lung tissue. Although several studies have shown the potential value of tumor volume reduction ratio (VRR) during RT for predicting survival in patients with NSCLC, this remains to be seen due to a lack of data for patients with SCLC [20–22].

We conducted this retrospective analysis of SCLC patients who received RT to investigate whether the volumetric reduction during treatment will have a possible impact on survival data. The goal was to determine additional prognostic factors and identify patients at increased risk for recurrence.

## Materials and methods

**Fig. 1:**
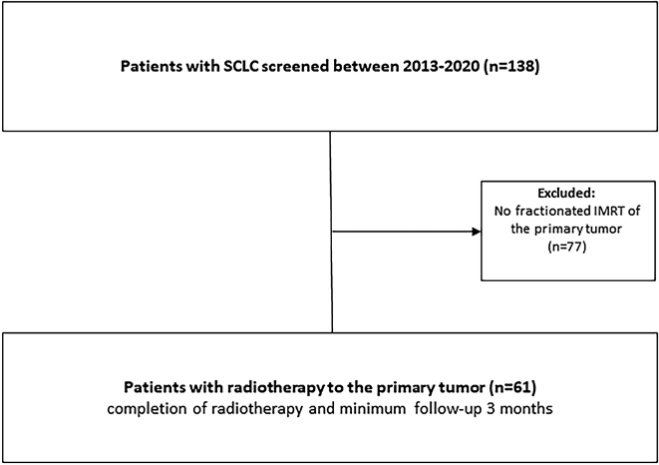
Flowchart of patient selection. *IMRT* intensity-modulated radiotherapy, *SCLC* with small-cell lung cancer

The present study included 61 patients who received fractionated IMRT of the primary tumor at the Radiation Oncology Department, University Hospital Muenster, in Muenster, Germany, between 2013 and 2020. The medical records of all patients were reviewed for tumor and treatment characteristics as well as for the clinical outcomes (Fig. 1). Almost all published data are based on the Veterans’ Administration Lung Study Group classification into limited or extensive disease. For better comparability, we also refer to a classification in “limited” or “extensive” and aim to identify a possible complement to already known prognostic markers [23]. Planning computed tomography scans (pCTs) were performed 2 weeks before starting RT (median, 7 days). The CT scans were acquired using the Aquilion CT system LB V3.38GR005 (Toshiba Medical Systems, Otawara, Japan), and CT-DICOM was created with 3‑mm slice thickness (120 KV, 100 mA and range: 600). Additionally, planning positron emission tomography (PET)-CT scans were performed for 28 (46%) patients. Imaging data were reviewed for the staging process, all according to the recently updated 8th edition of the TNM classification for malignant tumors rubric. Gross tumor volume (GTV) was manually contoured in the midventilation phase based on CT scans obtained at the time of CT simulation and new CT images obtained after 40 Gy. The GTV includes the primary tumor and the affected mediastinal lymph nodes. The planning tumor volume (PTV) included GTV with a 5–10-mm safety margin. In addition, 4D-CTs were performed to visualize and delineate tumor movement in a different respiratory phase. All patients were treated with the IMRT technique. A total of 59 patients (96%) received CTx (concurrent or sequential). All patients received at least a weekly kV CBCT scan (median 8 scans per patient). For each patient, GTV regression after 40 Gy (week 4–5) was calculated, with the patients separated into two groups based on median tumor regression value. The VRR was calculated as (GTV at 5th week − GTV at 1st week) / GTV at 1st week on a percentage scale. Following thoracic RT, 41 patients (67%) received prophylactic cranial irradiation (PCI) with 30.6 Gy (daily fraction was 1.8 Gy) within 4–8 weeks of thoracic RT. All patients had follow-up visits 2 months after completion of treatment, then every third month for 2 years, every sixth month for the following 3 years, and yearly after that. The date of progression was calculated from the locoregional recurrence (LRR) or distant metastasis.

### Statistical analysis

Time-dependent event curves were generated by the Kaplan–Meier method and compared with log-rank tests. The OS was calculated from the first diagnosis to the time of death. The PFS was calculated from RT initiation to the time of documented recurrence or death. Duration of locoregional control (LRC) was calculated from RT initiation to the time of documented local recurrence. Differences were considered statistically significant at a value of* p* < 0.05. Independent variables were first analyzed with univariate analysis. Variables shown by univariate analysis to be associated with LRC, PFS, or OS were entered into a Cox proportional hazards regression model for multivariate analysis. Chi-squared or Fisher exact tests were additionally performed in order to probe relationships between pairs of categorical variables. Finally, the two-sample *U *test was used to study the relationship between categorical and continuous variables. All statistical analyses were conducted with IBM SPSS Statistics 28.0 software (SPSS Inc., Chicago, IL, USA).

## Results

According to the TNM classification, 35 patients (57%), seven patients (12%), 11 patients (18%), and eight patients (13%) had T4, T3, T2, and T1 tumors, respectively. Regarding nodal involvement, 27 patients (44%), 19 patients (31%), seven patients (12%), and eight patients (13%) had cases of N3, N2, N1, and N0 disease, respectively. Additional patients’ characteristics are demonstrated in Table 1. Most patients (62%) received platinum-based CTx concurrently with RT. If the patient could not tolerate cisplatin, carboplatin-based CTx was administered. Six patients (10%) received atezolizumab immunotherapy in addition to cisplatin. By contrast, two patients (3%) did not receive CTx due to poor general conditions. The median number of total CTx cycles administered was six (range, 2–12 cycles).

**Table 1:** Patient and treatment characteristics

Characteristic	Value	Percentage/range
Mean age, years	65	35–82
Gender ratio	35 M: 26 F	–
**Stage according to**		
Extensive	14/61	23%
Limited	43/61	70%
Very limited	4/61	7%
**Tumor location**		
Upper lobe	33/61	54%
Lower lobe	14/61	23%
Hilar or mediastinum	14/61	23%
**Chemotherapy**	59/61	–
Concurrent	30/61	49%
Sequential	21/61	34%
Concurrent and sequential	8/61	13%
Med. number of CTx cycles	6	2–12
**Treatment parameters**		
*Med. radiation dose (range), Gy*	59.4	30–72.0
≤ 59.4 Gy	36/61	59%
> 59.4 Gy	25/61	41%
*Med. fraction dose (range), Gy*	1.8	1.8–3.0
*Med. GTV in pCT, cm*^*3*^	74	3–584
*VRR after 5 weeks of RT, %*	48%	3–95%
*Med. PTV, cm*^*3*^	510	38–1668
**Recurrence**	25/61	41%
Local only	5/61	8%
Distant only	7/61	12%
Local and distant	13/61	21%

*M* males, *F* females, *Med.* median, *pCT* planning CT, *VRR* volume reduction ratio, *CTx* chemotherapy, *PTV* planning tumor volume

**Fig. 2:**
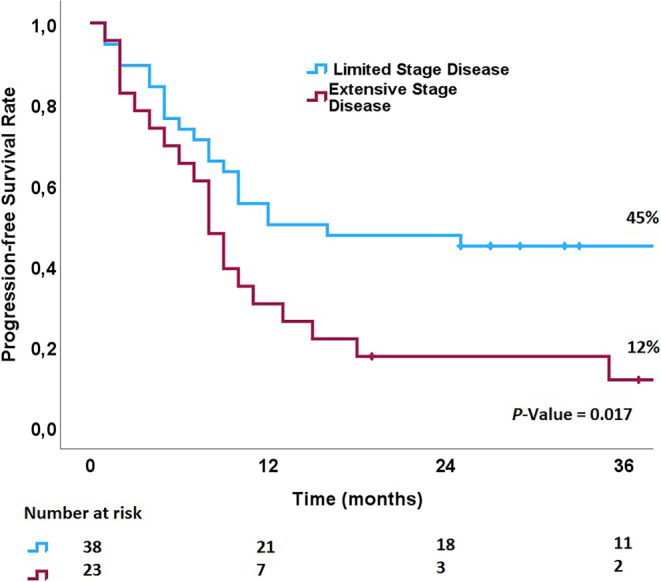
Kaplan–Meyer survival according to disease extension

The median follow-up time was 22 months (range, 3–116 months). For the whole cohort, the median OS and median PFS were 23 (95% CI: 16–30) and 14 months (95% CI: 9.4–18.6), respectively. The 2‑year OS and 2‑year PFS were 47% and 36%, respectively. The 2‑year LRC rate was 69%. Overall, 47 patients (77%) had limited-stage disease, and 14 (23%) had extensive-stage disease. In patients with very limited, with limited, and with extensive disease, the median OS was 24, 22, and 15 months, respectively (*p* = 0.3). There was longer PFS in patients with limited disease (12 vs. 8 months, *p* = 0.017; Fig. 2). No significant difference in LRC was found between the limited and extensive groups (*p* = 0.6). Regarding the delivered radiation dose, we could not detect any difference in relapse rate (*p* = 0.8), LRC (*p* = 0.9), PFS (*p* = 0.5), or OS (*p* = 0.98). Additionally, the initial GTV volume did not impact the LRC (*p* = 0.4), PFS (*p* = 0.7), or OS (*p* = 0.6).

### Reduction in tumor volume

All patients exhibited GTV reduction during RT with a median VRR of 48% (range, 3–95). Cisplatin-based CTx was associated with higher VRR compared to carboplatin-based CTx (49% vs. 40%, *P* = 0.3). We could not detect any significant difference in LRC (*p* = 0.34), PFS (*p* = 0.98), and OS (*p* = 0.8) between the group of patients who exhibited a high VRR (> median of 48%) and the group that exhibited a VRR ≤ 48%. Furthermore, in the subgroup analysis, we could not detect any survival difference between patients with limited and extensive SCLC who achieved higher VRR (*p* = > 0.05).

### Distant and local recurrences

At the end of this analysis, 40 patients (66%) had died. We detected tumor recurrences in 25 patients (41%), including 20 distant recurrences and 18 LRRs (with 13 patients having both). We found no significant association between VRR and risk of LRR evolution (*p* = 0.2). The relapse pattern was similar in patients with a high VRR and those with a low VRR (*p* = 0.7). Sites of distant relapse included the liver (*N* *=* 6), brain (*N* = 6), bone (*N* = 5), contralateral lung (*N* *=* 2), and adrenal gland (*N* *=* 1).

### Chemotherapy

The use of concurrent CTx was associated with a higher VRR (*p* = 0.05). Recurrence rates were similar regardless of whether cisplatin-based or carboplatin-based therapies were applied (*p* = 0.4) or the timing of CTx (*p* = 0.4). There was no significant association between the sequence of CTx (concurrent vs. sequential) and LRC (*p* = 0.4, respectively). However, the PFS (*p* = 0.009) and OS (*p* = 0.06) were longer with concurrent CTx. There was no significant association between the CTx regimen (cisplatin-based vs. carboplatin-based) and LRC or OS (*p* = 0.8 and 0.08, respectively), while the PFS was significantly longer following cisplatin-based CTx compared to carboplatin-based CTx (*p* = 0.01). Although an increased number of CTx cycles showed an improvement in local-regional control (*p* = 0.002), no significant improvement in PFS (*p* = 0.14) or OS (*p* = 0.24) was observed.

### Cox proportional hazards model

Age at the time of RT, tumor extension, VRR, use of CTx, number of CTx cycles, and prophylactic WBI delivery were included in a Cox proportional hazard model (Table 2). In the multivariate analysis, age remained related to PFS (*p* = 0.07) and OS (*p* = 0.04), the number of CTx cycles remained related to LRC (*p* = 0.002). Additionally, the prophylactic WBI remained related to PFS (*p* = 0.02).

**Table 2:** Univariate and multivariate analyses for LRC, PFS, and OS (*N* = 61)

Risk factor	LRC		PFS		OS	
	HR	*p*	HR	*p*	HR	*p*
*Univariate model*						
Age (years)	1.006	0.8	1.037	**0.05**	1.039	**0.04**
Limited vs. extensive disease	0.739	0.6	0.555	**0.09**	0.670	0.3
VRR	0.989	0.3	1.000	0.9	0.997	0.7
CTx concurrent vs sequential	0.628	0.4	0.457	**0.01**	0.542	**0.06**
Number of CTx cycles	1.325	**0.002**	1.108	0.14	1.096	0.24
Prophylactic WBI	0.800	0.7	0.564	**0.07**	0.649	0.2
*Multivariate model*						
Number of CTx cycles	1.325	**0.002**	–	–	–	–
Age (years)	–	–	1.034	0.07	1.040	**0.04**
Prophylactic WBI	–	**–**	0.466	**0.02**	–	**–**

*LRC* locoregional control, *PFS* progression-free survival, *OS* overall survival, *HR* hazard ratio, *RT* radiotherapy, *VRR* volume reduction ratio, *CTx* chemotherapy

## Discussion

Inter- and intrafraction tumor monitoring is crucial for patients with lung cancer, as volumetric changes occur during RT. Repeated adaptive planning allows for adequate PTV coverage and reduced damage to normal lung tissue while sparing organs at risk [24–27]. Regarding volume regression, patients with SCLC have been shown to experience more volumetric regression to RCT than patients with NSCLC [20]. Several studies have shown that CT-based tumor volume is an independent prognostic factor, as is volume reduction during RT [20, 28, 29]. To the best of our knowledge, no data on the prognostic impact of postradiation volume changes in patients with SCLC have been reported.

In this study, we analyzed the potential value of tumor volume regression during irradiation of patients with SCLC. We could not detect any significant impact on the outcome of initial tumor volume and volumetric changes during RT. Recently a study reported that pretherapeutic CT-based radiomics features predict treatment outcomes following RCT of SCLC patients [30]. Kamran et al. [30] evaluated various radiomics features in 105 SCLC patients with limited disease. In accordance with our data, GTV appeared to be unrelated in terms of prognosis; however, the authors found that the maximum 3D diameter of the primary tumor correlated significantly with outcomes. Possible clarifications that GTV reduction did not affect prognosis could be the difference in timing between initial diagnosis and initiation of therapy, the timing of CTx, and the heterogeneous patient population. Future analyses would need to select a prospective study with uniform baseline and staging. Although a recent meta-analysis showed that conventional fractionation remains an acceptable option, the optimal dose and fractionation of thoracic RT remains controversial. In vitro studies have demonstrated the remarkable radiosensitivity of SCLC cell lines, even when exposed to low doses of radiation. This characteristic prompted investigations of hyperfractionated accelerated irradiation compared to conventional fractionated RT with a total dose of 45 Gy. Initial results suggested the superiority of twice-daily accelerated irradiation. However, follow-up studies showed no significant difference in 5‑year survival rates. An international randomized trial compared higher-dose conventional irradiation (66 Gy/2 Gy) with hyperfractionated irradiation (45 Gy/2 × 1.5 Gy) in SCLC patients. The study reported no significant difference in OS between the two treatment arms, with similar levels of treatment-related toxicity [31–33]. However, once-daily RT is still the most commonly used regimen in clinical practice due to logistical issues, patient inconvenience, institutional expertise and the lack of statistically significant inferiority in survival difference and toxicity between the twice-daily and once-daily patients in the CONVERT trial. Therefore, both treatment regimens can currently be recommended as viable options for patients, although recent data may suggest a trend toward twice-daily irradiations [19, 34]. Nevertheless, given the existing controversies, it would be interesting for future studies to investigate the impact of shortened overall treatment time on patient outcomes in terms of VRR, as a higher VRR would be expected in this context.

Regarding CTx administration, concurrent CTx correlated with higher VRR (*p* = 0.05) than sequential CTx. We also detected a higher VRR with cisplatin based CTx. In addition, we could not detect any significant association between the CTx regimen (cisplatin-based vs. carboplatin-based) and LRC or OS. However, an increased number of CTx cycles demonstrated a significant impact on LRC (*p* = 0.002) and the PFS showed a significant improvement with cisplatin-based CTx (*p* = 0.01). The existing question of which of the two platinum-based CTx is better for patients cannot be answered precisely in this analysis. In addition, patients who received cisplatin-based CTx experienced higher VRR. According to a meta-analysis of randomized trials, a cisplatin-based regimen should be the first-choice CTx combination. Carboplatin-based protocols might be recommended if cisplatin is contraindicated [35]. An advantage of both regimens over possible alternatives is their unrestricted applicability in concurrent RCT. In addition, the studies known to date show that concurrent RCT improves patient survival [36]. This is reflected in our data with a significantly longer PFS (*p* = 0.009) and a trend toward a better OS (*p* = 0.06) than sequential CTx. We could not detect any impact of concurrent CTX compared with sequential CTx on LRC. This study is limited by its retrospective nature and relatively small size. However, despite these limitations, the results are intriguing and could serve as a basis for further investigation, particularly regarding daily fractions and irradiation dose [37]. Biological and radiomic data are needed to identify SCLC patients who may benefit from repeated radiation plan adaptations and possibly dose escalation [38].
